# The ratio of respiratory rate to diaphragm thickening fraction for predicting extubation success

**DOI:** 10.1186/s12890-023-02392-w

**Published:** 2023-04-04

**Authors:** Dararat Eksombatchai, Chalermwut Sukkratok, Yuda Sutherasan, Detajin Junhasavasdikul, Pongdhep Theerawit

**Affiliations:** 1 Division of Pulmonary and Pulmonary Critical Care Medicine, Department of Medicine, Faculty of Medicine Ramathibodi Hospital, Mahidol University, 270 Ramathibodi Hospital, Rama VI Road, Bangkok, 10400 Ratchathewi Thailand; 2 Division of Critical Care Medicine, Department of Medicine, Faculty of Medicine Ramathibodi Hospital, Mahidol University, 270 Ramathibodi Hospital, Rama VI Road, Bangkok, 10400 Ratchathewi Thailand

**Keywords:** Diaphragm ultrasound, Ultrasound, Extubation, Weaning, Rapid shallow breathing index, Diaphragm thickening fraction, Diaphragmatic excursion

## Abstract

**Background:**

Several parameters are used to predict successful extubation but their accuracy varies among studies. We hypothesized that combining conventional and diaphragmatic parameters would be more effective than using just one. Our primary objective was to evaluate the performance of the respiratory rate in relation to the diaphragm thickening fraction (RR/DTF) ratio to predict the success of extubation.

**Methods:**

We enrolled 130 adult patients who required invasive mechanical ventilation, planned to be extubated, and used a spontaneous breathing trial (SBT) in the intensive care unit from July 2020 to April 2022. We measured the conventional parameters and the diaphragmatic parameters 2 h after SBT. The RR/DTF was calculated by dividing the respiratory rate (RR) by the diaphragm thickening fraction (DTF). The definition of weaning success is successful extubation within 48 h.

**Results:**

Of 130 patients, 8 patients (6.2%) were reintubated within 48 h. The RR/DTF was significantly lower in the successful extubation group than in the extubation failure group (right hemidiaphragm; 0.47 (0.33–0.64) vs 1.1 (0.6–2.32), *p* < 0.001 and left hemidiaphragm; 0.45 (0.31–0.65) vs 0.78 (0.48–1.75), *p* < 0.001). The right RR/DTF using a cut-off point at ≤ 0.81 had a sensitivity of 87.7%, a specificity of 75%, and areas under the receiver operating characteristic curve (AUROC) of 0.762 for predicting successful extubation (*p* = 0.013). The sensitivity, specificity, and AUROC for predicting extubation success of right DTF at a cut-off point of ≥ 26.2% were 84.3%, 62.5%, and 0.775, respectively (*p* = 0.009).

**Conclusion:**

The RR/DTF ratio is a promising tool for predicting extubation outcome. Additionally, using RR/DTF was more reliable than conventional or diaphragmatic parameters alone in predicting extubation success.

**Supplementary Information:**

The online version contains supplementary material available at 10.1186/s12890-023-02392-w.

## Background

Predicting liberation from mechanical ventilation (MV) has long been a crucial clinical issue. Extubation failure was associated with an increased risk of death and an extended ICU stay [1]. Currently, parameters such as the rapid shallow breathing index (RSBI), vital capacity (VC), and maximal inspiratory pressure (PI_MAX_) are routinely used to predict extubation failure from MV. However, the results of these parameters in predicting weaning outcomes vary across studies [2–5].

The ratio of respiratory rate to tidal volume (RR/VT), known as the RSBI, is one of the most widely used predictors of weaning outcomes. However, some previous studies showed that RSBI is not a reliable predictor of extubation success [5–7]. These results may be because RSBI does not specifically measure diaphragmatic function. The diaphragm is the main respiratory muscle. It has been demonstrated that MV accelerates diaphragmatic atrophy [8]. Diaphragm dysfunction (DD) is common in critically ill patients especially in patients with respiratory failure who require MV [9]. Furthermore, DD is related to weaning failure [10]. Therefore, parameters that do not directly measure diaphragmatic function might be poor predictors of extubation outcomes.

Assessment of diaphragmatic function by the twitch magnetic phrenic nerve stimulation or measurement of transdiaphragmatic pressure with esophageal balloons is costly and invasive. Bedside ultrasound is increasingly performed in a critical care setting and may be of great utility for this purpose because it is non-invasive, widely available, and allows real-time evaluation of diaphragmatic movement. To determine diaphragmatic function, ultrasound can be used to obtain diaphragm thickening fraction (DTF), diaphragmatic excursion (DE), and time to peak inspiratory amplitude (TPIA) during contraction [10–14].

Spadaro et al. revealed that replacing VT with DE in the RSBI, named D-RSBI (RR/DE), had better diagnostic accuracy for predicting weaning outcomes than traditional RSBI [15]. The RSBI reflects the function of all inspiratory muscles and accessory muscles. In case of diaphragmatic dysfunction, the other inspiratory and accessory muscles will serve to preserve tidal volume (VT), which is used to calculate RSBI. However, those groups of muscles can only temporarily replace the diaphragm function because the accessory muscles are weaker and more fatigable than the diaphragm [16, 17]. These muscles will not be able to sustain adequate ventilation. Thus, we hypothesized that modifying RSBI by replacing VT with a diaphragmatic ultrasound parameter would provide a superior predictor to predict extubation outcome more than the conventional parameter or diaphragmatic parameter alone.

Several studies showed that DTF was more reliable than DE in terms of predicting weaning outcomes, with higher sensitivity and specificity [18, 19]. We hypothesized that RR/DTF would outperform RR/VT and RR/DE. Thus, the objective of this study was to determine the performance of RR/DTF ratio and to compare its accuracy with diaphragmatic and conventional parameters in predicting the success of extubation within 48 h.

## Methods

This prospective cross-sectional study was conducted at the tertiary-care hospital between July 2020 and April 2022. The study was approved by the Ethics Clearance Committee on Human Rights Related to Research Involving Human subjected, Faculty of Medicine Ramathibodi Hospital, Mahidol University (approval no. MURA2020/881).

Patients aged 18 years and older were enrolled in this study from the medical and surgical intensive care units (ICU). The inclusion criteria were as follows: 1) patients with acute respiratory failure caused by medical or postoperative conditions who had been receiving MV for more than 48 h and tolerated 2 h spontaneous breathing trial (SBT); 2) readiness for weaning from MV as defined according to a local guideline (Additional file1: S1), including recovery from the cause of respiratory failure, hemodynamic stability in the absence of vasopressors, and no administration of neuromuscular blocking agents or sedative drugs for more than 24 h prior to enrollment with a Richmond agitation-sedation scale (RASS) score between -1 and + 1. All patients or their relatives were able to give written informed consent prior to enrollment.

Exclusion criteria included a history of neuromuscular disease or thoracic surgery, pneumothorax, presence of ascites, tracheostomized patients, and poor image quality.

Baseline characteristics were obtained including age, sex, body mass index (BMI), length of MV until SBT, underlying disease, cause of respiratory failure, and laboratory findings.

### Measurements

All participants underwent a SBT by either using pressure support ventilation (PSV) with pressure support of 5 cmH_2_O and positive end-expiratory pressure (PEEP) of 5 cmH_2_O or a T-piece system with oxygen support to achieve oxygen saturation of ≥ 92%.

The decision to start weaning, extubation or reintubation was made based on the attending physician's discretion following local guidelines. Daily, patients were assessed by the attending physician for weaning readiness using local criteria (Additional file1: S1). Criteria for failed SBT are shown in Additional file1: S2. Investigators were informed when the ICU attendings decided to extubate. The attending physicians were blinded to the ultrasound results. Following extubation, patients who were considered to be at high risk of extubation failure may be extubated directly to preventative noninvasive ventilation (NIV) or high-flow nasal cannula based on the decision of the attending physicians. Patients at high risk of extubation failure include patients with hypercapnia, COPD, congestive heart failure, or high oxygen requirement. Reintubation was decided by the attending physicians. In our clinical practice, we generally use the local criteria for assessing extubation failure as shown in Additional file 1: S3.

Exhaled VT, RR, RSBI, VC, PI_MAX_, and diaphragmatic parameters were measured in all participants at 2 h after the SBT.

We used a hand-held Wright respirometer (Ferraris Medical Ltd., Herford, Hertfordshire, England) to measure minute ventilation. VT was calculated as minute ventilation divided by RR. The RSBI was obtained by dividing RR (breaths/min) with VT (litres). Measurement of VC was performed in the upright position after measurement of minute ventilation. PI_MAX_ was obtained by occluding the airway at end-expiration through a unidirectional valve, a calibrated device attached to the end of an endotracheal tube (Instrument Industry, Inc., Bethel Park, PA, USA). PI_MAX_ was the most negative pressure documented after airway occlusion when patients were instructed to take a maximal inspiratory effort against the closed valve [20].

Transthoracic ultrasonography was performed at the bedside by a well-trained pulmonary physician using SonoSite M-Turbo (SonoSite Inc., Bothell, WA, USA). All examinations were carried out with patients in a semi-recumbent position with the head of the bed at 30–45 degrees. We obtained diaphragmatic ultrasound values from three consecutive tidal breaths on each side of the hemidiaphragm, and average values were used for analysis.

**Fig. 1:**
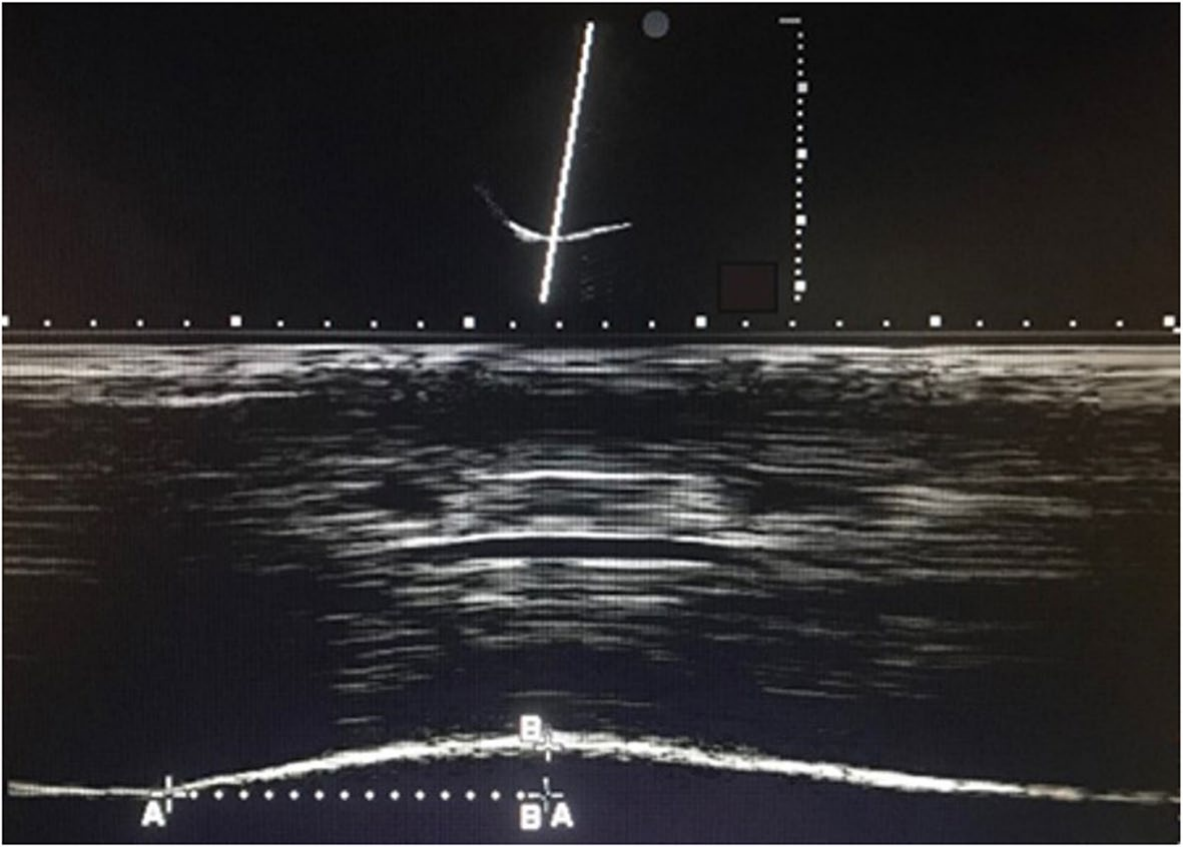
Image demonstrating measurement of the diaphragmatic inspiratory excursion (DE) and time to peak inspiratory amplitude (TPIA) in M-mode ultrasound. A-A is the TPIA. B-B is the DE

The diaphragmatic inspiratory excursion (DE) and time to peak inspiratory amplitude of the diaphragm (TPIA) of each hemidiaphragm were measured in M-mode using a 1- to 5-MHz ultrasound transducer during tidal breathing. The ultrasound probe was placed at the junction of the mid-clavicular line and subcostal margin or intercostal space in which the ultrasound beam paralleled the direction of diaphragmatic movement. Using M-mode tracing, the normal diaphragm movement towards the probe during inspiration was recorded as an upward motion. The TPIA was defined as the time from the beginning of diaphragmatic contraction to the maximal amplitude of diaphragmatic excursion (Fig. 1).

**Fig. 2:**
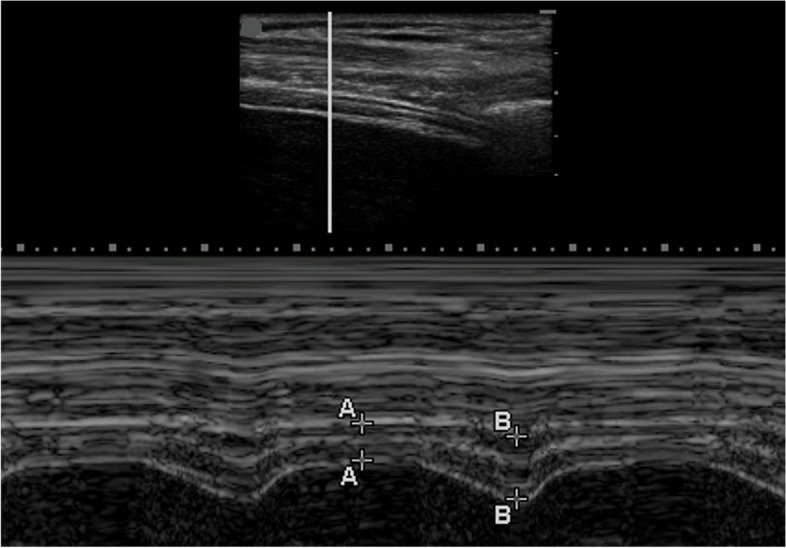
Image at zone of opposition for measurement of diaphragmatic thickness (DT). A-A is the DT at end expiration, and B-B is the DT at end inspiration

Diaphragmatic thickness (DT) was subsequently measured at the zone of apposition, which is the area of the diaphragm attached to the rib cage between the eighth and tenth intercostal spaces. The DT was measured at both end inspiration and end expiration using a 6–13 MHz linear ultrasound transducer in M-mode (Fig. 2). The diaphragm thickening fraction (DTF) percentage was calculated with the following formula: thickness at the end of inspiration minus thickness at the end of expiration, divided by thickness at the end of expiration, then multiplied by 100 [21]. The RR/DTF was calculated by dividing the respiratory rate (RR) by the DTF. The RR/DE was calculated by dividing the respiratory rate (RR) by the DE.

### Outcomes

The primary outcome was the diagnostic accuracy of the RR/DTF for predicting successful extubation. Extubation failure was defined as the requirement of reintubation within 48 h after extubation.

The secondary outcome was to compare the diagnostic accuracy of the RR/DTF with diaphragmatic parameters (DE, DTF, TPIA) and conventional parameters (RSBI, VC, PI_MAX_) for predicting successful extubation.

### Statistical analysis

The sample size was calculated with an area under the receiver operating characteristic curve (AUROC) of greater than 0.80 as acceptable diagnostic accuracy. Accordingly, assuming that the null hypothesis for AUROC was 0.5 at a power of 80%, a Type I error of 0.05, and a prevalence of 9.7% reintubation within 48 h [13], a minimal sample size of 83 patients was required in this study.

The patients were categorized into two groups based on the primary outcome. The unpaired Student's t-test was used to compare continuous variables and the Chi-square test was used to compare categorical variables. Data were presented as mean ± standard deviation for continuous variables with a normal distribution and median with interquartile range for variables without normal distribution.

The variables associated with the primary and secondary outcomes were then analyzed with receiver operating characteristic (ROC) curves to determine their performance. Sensitivity and specificity were also analyzed to determine appropriate cut-off values for each parameter.

We analyzed inter-operator variability of all diaphragmatic ultrasound parameters in 10 cases by intraclass correlation coefficient. Statistical analysis was conducted using IBM SPSS statistics for Windows, version 22.0 (IBM Corp., Armonk, NY, USA).

## Results

In total, 138 patients were enrolled in our study. Eight patients were excluded due to poor image quality. A total of 130 patients were included for analysis. The mean age was 68.1 ± 16.1 years; 78 patients (60%) were male. One hundred and twenty-two patients (93.8%) were successfully extubated without reintubation within 48 h. The baseline characteristics, comorbidities, the reasons for intubation, and laboratory findings of the extubation success and failure groups were shown in Table 1.

**Table 1:** Baseline characteristic between extubation success and extubation failure groups

Variables	Total (*n* = 130)	Extubation success (*n* = 122)	Extubation failure (*n* = 8)	*p*-value
Demographic data				
Age, mean ± SD, years	68.1 ± 16.1	68.1 ± 16.2	69.1 ± 15.6	0.86
Sex (n/%)				0.88
Female	520 (40)	49 (40.2)	3 (37.5)	
Male	78 (60)	73 (59.8)	5 (62.5)	
BMI, mean ± SD, kg/m^2^	22.3 ± 5.1	22.2 ± 5.1	24.4 ± 6.7	0.28
APACHE II Score	18.1 ± 7.4	18.1 ± 7.6	18.4 ± 3.4	0.95
Admission ward				0.82
Medical patients, n (%)	110 (85)	103 (84.4)	7 (87.5)	
Surgical patients, n (%)	20 (15)	19 (15.6)	1 (12.5)	
Comorbidity, n (%)				
Hypertension	77 (59.2)	72 (59)	5 (62.5)	0.85
Diabetes	45 (34.6)	42 (34.4)	3 (37.5)	0.86
Chronic kidney disease	41 (31.5)	39 (32)	2 (25)	0.68
Ischemic heart disease	30 (23.1)	30 (24.6)	0	0.11
COPD	15 (11.5)	15 (12.3)	0	0.29
Hypothyroid	8 (6.2)	8 (6.6)	0	0.46
Adrenal insufficiency	3 (2.3)	3 (2.5)	0	0.65
Asthma	3 (2.3)	3 (2.5)	0	0.65
Reason for intubation, n (%)				
Neurological disease	22 (16.9)	21 (17.2)	1 (12.5)	0.73
Septic shock	29 (22.3)	26 (21.3)	3 (37.5)	0.29
Pneumonia	27 (20.8)	24 (19.7)	3 (37.5)	0.23
Heart disease	12 (9.2)	12 (9.8)	0	0.35
COPD exacerbation	5 (3.8)	5 (4.1)	0	0.56
Post-operative conditions	22 (16.9)	21 (17.2)	1 (12.5)	0.73
Laboratory, mean ± SD				
Sodium (mmol/L)	137.6 ± 5.5	137.7 ± 5.5	135.5 ± 6.2	0.28
Potassium (mmol/L)	4.0 ± 0.5	4.0 ± 0.5	3.8 ± 0.3	0.19
Bicarbonate (mmol/L)	23.4 ± 4.0	23.4 ± 4.1	23.7 ± 4.0	0.86
Magnesium (mmol/L)	8.1 ± 0.7	2.0 ± 0.4	2.0 ± 0.5	0.85
Phosphate (mmol/L)	2.0 ± 0.4	4.0 ± 3.7	3.2 ± 0.5	0.56
Calcium (mmol/L)	4.0 ± 3.5	8.1 ± 0.7	8.3 ± 0.6	0.47
Albumin (g/L)	25.7 ± 6.2	25.6 ± 6.3	26.1 ± 3.3	0.84
Creatinine (mmol/L), median (IQR)	1.1 (0.7–2.3)	1.1 (0.7–2.3)	0.9 (0.6–2.4)	0.81
Hematocrit (%)	31.0 ± 6.6	30.9 ± 6.7	31.9 ± 6.0	0.69
Length of MV until SBT (days), median (IQR)	3.7 (2.4–6.5)	3.4 (2.3—6.4)	6.9 (4.8—20.7)	0.04
Total LOS (days), median (IQR)	17.8 (9.2 – 34)	15.8 (8.8—30)	32.4 (20.7—49.6)	0.08
ICU LOS (days), median (IQR)	7.8 (4.2 – 15.7)	7.3 (4.1—13.8)	17.7 (13.6—31.2)	0.02

*APACHE II* Acute Physiology and Chronic Health Evaluation II, *COPD* chronic obstructive airway disease, *ICU* intensive care unit*, LOS* length of stay*, MV* mechanical ventilation, *SBT* spontaneous breathing trail*, IQR* interquartile range*, CI* confidence interval, *SD* standard deviation

All diaphragmatic parameters obtained from the right and left hemidiaphragm were compared, as shown in Table 2. The mean diaphragmatic excursion of the right and left hemidiaphragm was 1.6 ± 0.7 cm and 1.5 ± 0.7 cm (*p* = 0.15). The mean diaphragmatic thickening fraction of the right and left hemidiaphragm was 41.1 ± 17.6% and 42.6 ± 18.4% (*p* = 0.25). The mean TPIA of the right hemidiaphragm was statistically significantly higher than the left hemidiaphragm (1.2 ± 0.4 s vs. 1.1 ± 0.4 s, *p* < 0.001).

**Table 2:** Comparison between right and left diaphragmatic parameters

Ultrasonographic parameters	Right	Left	*p*-value
Diaphragmatic excursion (cm)	1.6 ± 0.7	1.5 ± 0.7	0.15
Thickness inspiration (mm)	3.2 ± 1.1	3.3 ± 1.1	0.27
Thickness expiration (mm)	2.3 ± 0.8	2.3 ± 0.8	0.83
Diaphragm thickening fraction (DTF) (%)	41.1 ± 17.6	42.6 ± 18.4	0.25
Time to peak inspiratory amplitude (TPIA) (second)	1.2 ± 0.4	1.1 ± 0.4	< 0.001

All data are present as mean ± SD

RR/DTF from both hemidiaphragm was significantly lower in the extubation success group than in the extubation failure group. The RR/DTF of the right hemidiaphragm in both groups were 0.47 (0.33–0.64) vs 1.1 (0.6–2.32), *p* < 0.001, and that of the left hemidiaphragm were 0.45 (0.31–0.65) vs 0.78 (0.48–1.75), *p* < 0.001. As shown in Table 3, bilateral RR/DE was significantly lower in the extubation success group than in the extubation failure group. Bilateral diaphragmatic thickness fraction and right diaphragmatic excursion were significantly higher in the extubation success than in the failure group. RSBI was lower in the extubation success than in the failure group, 53.1 ± 24 vs. 80.6 ± 52.2, *p* = 0.005. The vital capacity (VC) significantly differed between patients who succeeded and failed extubation, 987.8 ± 361.3 vs. 720 ± 365.4 ml*, p* = 0.045.

**Table 3:** Comparison of parameters between extubation success and extubation failure groups

Parameters	Extubation success (*n* = 122)	Extubation failure (*n* = 8)	*p*-value	95% CI
**Combination of RR and diaphragmatic parameters**				
RR/DTF (breaths/min/%), median (IQR)				
Right	0.47 (0.33–0.64)	1.1 (0.6–2.32)	< 0.001	-1.36 to -0.56
Left	0.45 (0.31–0.65)	0.78 (0.48–1.75)	< 0.001	-1.29 to -0.52
RR/DE (breaths/min/cm), median (IQR)				
Right	12.06 (8.04–18.87)	18.15 (14.02–27)	< 0.001	-28.66 to -8.34
Left	12.16 (8.25–20)	15.8 (13.02–31.07)	0.002	-29.33 to -6.83
**Diaphragmatic parameters**				
Diaphragmatic thickness fraction (%), mean ± SD				
Right	42.2 ± 17.4	25.5 ± 14.6	0.009	4.20 to 29.09
Left	43.6 ± 18.2	28.7 ± 15.3	0.025	1.86 to 27.96
Diaphragmatic inspiratory excursion (cm), mean ± SD				
Right	1.6 ± 0.7	1.1 ± 0.4	0.039	0.03 to 1.02
Left	1.6 ± 0.7	1.2 ± 0.5	0.103	-0.08 to 0.85
Time to peak inspiratory amplitude of diaphragm (second), mean ± SD				
Right	1.2 ± 0.4	1.0 ± 0.4	0.082	-0.03 to 0.49
Left	1.1 ± 0.4	0.9 ± 0.3	0.052	-0.002 to 0.50
**Conventional parameters**				
RSBI (breaths/min/L), median (IQR)	50 (36–66)	70 (50–97)	0.005	-46.50 to -8.53
VC (ml), mean ± SD	987.8 ± 361.3	720.0 ± 365.4	0.045	6.59 to 528.91
PI_MAX_ (mmHg), mean ± SD	37.1 ± 15.5	30.4 ± 9.3	0.23	-4.28 to 17.66

*DTF* diaphragm thickening fraction, *DE* diaphragmatic excursion, *VC* vital capacity, *PI*_*MAX*_ maximal peak inspiratory pressure, *RSBI* rapid shallow breathing index, *RR* respiratory rate, *IQR* interquartile range, *CI* confidence interval, *SD* standard deviation

**Fig. 3:**
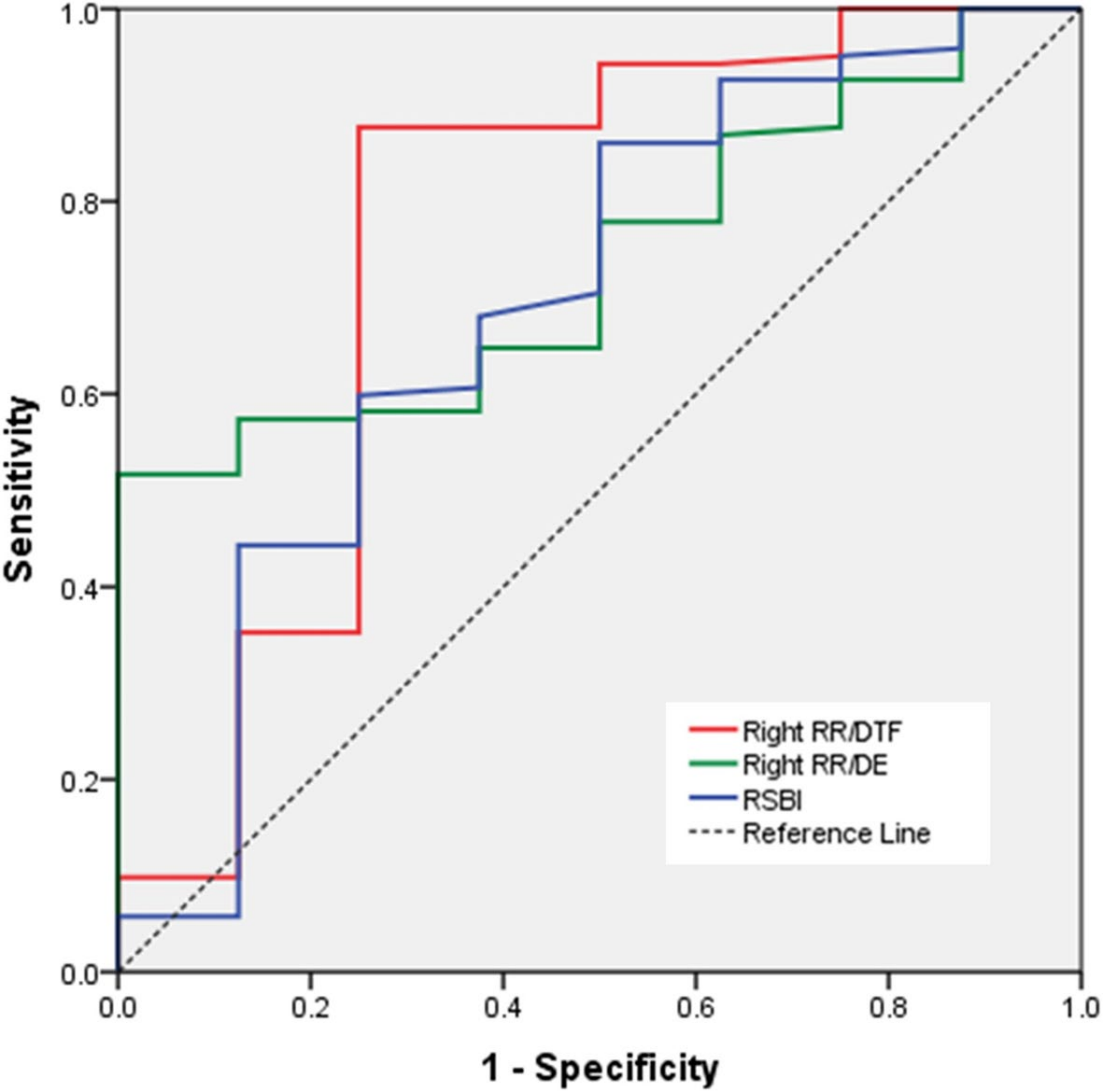
The receiver operating characteristic curve for predicting of extubation success of right RR/DTF, right RR/DE, and RSBI

The sensitivity, specificity, and AUROC for predicting of extubation success of RR/DTF, RR/DE, DTF, DE, TPIA, VC, and RSBI are shown in Table 4. The right RR/DTF provided the highest sensitivity and specificity compared to other parameters. To predict extubation success, the RR/DTF of the right hemidiaphragm with a threshold value of ≤ 0.81 provided a sensitivity of 87.7% and specificity of 75%, AUROC = 0.762. The receiver operating characteristic curve for predicting of extubation success of right RR/DTF, right RR/DE, and RSBI is demonstrated in Fig. 3.

**Table 4:** Sensitivity, specificity, positive predictive value, negative predictive value, and area under the receiver operating characteristic curve of parameters for predicting of extubation success

Test result variables	Cut-off value	Sensitivity	Specificity	PPV	NPV	*P*-value	AUROC
**Combination of RR and diaphragmatic parameters**							
Right RR/DTF	≤ 0.81	87.7	75	98.2	28.6	0.013	0.762
Left RR/DTF	≤ 0.75	86.1	62.5	96.3	19	0.020	0.746
Right RR/DE	≤ 20.45	77.9	50	96	12.9	0.025	0.737
Left RR/DE	≤ 15.72	65.6	62.5	96.4	10.6	0.057	0.701
**Diaphragmatic parameters**							
Right DE	≥ 1.05	78.5	50	96	12.9	0.029	0.730
Right DTF	≥ 26.2	84.3	62.5	97.1	20	0.009	0.775
Left DTF	≥ 27	82.6	50	96.2	15.4	0.033	0.725
**Conventional parameters**							
RSBI	≤ 60.5	70.5	50	95.6	10	0.069	0.692
Vital capacity	≥ 616.5	85.1	62.5	97.2	21.7	0.028	0.732

*RR* respiratory rate*, DTF* diaphragm thickening fraction*, DE* diaphragmatic excursion*, RSBI* rapid shallow breathing index, *PPV* positive predictive value, *NPV* negative predictive value, *AUROC* area under the receiver operating characteristic curve

The inter-operator variability analysis of DE, TPIA, and DTF between the two operators revealed an intraclass correlation coefficient of 0.99 (95%CI 0.98–0.99), 0.98 (95%CI 0.9–0.99), and 0.97 (95%CI 0.89–0.99); *p* < 0.001, respectively.

## Discussion

This study demonstrated that the ratio of RR/DTF indicated good predictive performance for successful extubation. With the threshold value of ≤ 0.81, the right RR/DTF exhibited a sensitivity of 87.7% and a specificity of 75% for predicting extubation success. The right RR/DTF had higher sensitivity and specificity in predicting extubation success than other parameters.

We found that DTF had comparable sensitivity to RR/DTF in predicting extubation success. However, RR/DTF had a higher specificity than DTF. The most likely explanation is that RR/DTF incorporates RR, which represents overall respiratory muscle function [22], and DTF, which represents a diaphragmatic function, making it superior to the diaphragmatic parameter alone.

We found that RR/DTF was better than RSBI (RR/VT) for predicting extubation success. The superiority of RR/DTF may be attributed to the importance of the diaphragm muscle. RSBI uses division by VT, which is the result of respiratory muscles defeating the inspiratory load [23] to maintain adequate VT. However, the fact that respiratory muscles can overcome inspiratory load may reflect the overall function of the diaphragm and other respiratory muscles. Declined diaphragmatic functions can be compensated by other respiratory muscles. As a result, RSBI is inferior to RR/DTF, a parameter that directly evaluates the diaphragm function. Thus, replacing VT with DTF or DE would better predict the success of extubation.

This study also found that RR/DTF was better than RR/DE for predicting extubation success. This finding might be explained by the fact that DTF reflects the work of breathing [24]. Two physiologic studies discovered a correlation between DTF and esophageal pressure–time product and diaphragmatic pressure–time product [24, 25]. They demonstrated that diaphragm thickening accurately predicted changes in inspiratory muscle effort in response to changes in a support level. However, the diaphragmatic excursion was not correlated to any index of muscle effort under varying levels of muscle loading [25]. Therefore, DE may not be a reliable indicator of diaphragmatic contractile activity and inspiratory effort during PSV. Our study assessed diaphragmatic parameters in some patients while in PSV mode, which could explain why RR/DTF outperformed RR/DE in this study.

The cut-off value of DTF for predicting extubation success in our study is close to previous studies. In this study, the DTF cut-off value of ≥ 26.2% showed AUROC 0.775. DiNino et al. demonstrated that the AUROC of the right DTF for predicting extubation success was 0.79 when using a cut-off value of ≥ 30% [11]. According to Baess et al., while applying a cut-off value of ≥ 30%, the right DTF, AUROC for successful extubation prediction was 0.655 [26]. Differently, Ferrari et al. reported a cut-off value of 36% with an AUROC of 0.948 [21]. This discrepancy could be explained by method heterogeneity, such as differences in patient populations, definitions of weaning failure, and the method of diaphragmatic ultrasound.

There was no difference between the right and left hemidiaphragmatic parameters except the TPIA. However, the difference in the TPIA between 1.2 s of the right hemidiaphragm and 1.1 s of the left hemidiaphragm was considered clinically insignificant. Therefore, in patients without unilateral diaphragmatic weakness, physicians can use the right-sided parameters alone if the left hemidiaphragm cannot be assessed. Furthermore, we observed that the right diaphragmatic parameters were more accurate than the left ones in predicting extubation success. This finding could be related to the difficulty in assessing the left diaphragm due to the smaller acoustic window of the spleen and the gas interposition in the stomach and intestine [14]. As a result, the measurement of the left diaphragm may be less accurate.

Many studies revealed that integrating multiple parameters improves accuracy for predicting successful extubation compared to using only one parameter [27, 28]. Our study demonstrated a new combining parameter that is reliable and easy to use. We also demonstrated a strong inter-observer correlation. Nevertheless, there are some limitations to this study. First, our study has a low rate of extubation failure (6.2%) compared to other studies [11, 13] which may have an impact on the result of the study. Second, this study included both medical and postoperative patients. As a result, we cannot apply the outcomes to specific diseases. Third, we performed a diaphragmatic ultrasound on patients using a T-piece or PSV mode, which may affect the study's findings because ultrasound results may vary according to the method of SBT. A previous study in patients during post-extubation, the median value of DTF and transdiaphragmatic pressure–time product (PTPdi) was significantly lower in spontaneous breathing than during NIV at the pressure support level of 5 cmH2O [24]. Another study showed that patients on MV with a pressure support level of 0 had a significantly lower value of DTF and PTPdi compared to a pressure support level of 5 [25]. Thus, the difference in SBT method in our study may have an effect on the value of DTF and work of breathing. As a result, research that is restricted to one mode of SBT is necessitated. Fourth, only patients with hemodynamic stability in the absence of vasopressors and no sedative or neuromuscular blocking agents administered for more than 24 h before extubation were included. We used these criteria to standardize the included population. However, in real-world practice, minimal vasopressor with hemodynamic stability and discontinuation of sedation less than 24 h with adequate mentation before considering extubation are accepted [29]. As a result, these inclusion criteria may not be representative of real-world practice. Fifth, the extubation failure group had a longer length of MV until SBT than the extubation success group. The results of the diaphragmatic parameters may be affected by the longer MV time until SBT because prolonged MV results in diaphragmatic contractile dysfunction [30]. However, in our intensive care unit, we generally use a local guideline daily to determine whether each ICU patient is ready to wean so that time to SBT is not delayed.

## Conclusion

In conclusion, this study exhibits a new parameter to predict successful extubation. The ratio of RR/DTF demonstrated good performance for predicting the success of extubation, and RR/DTF was superior to RSBI (RR/VT), RR/DE, and DTF in predicting extubation success. The better performance of RR/DTF may be explained by the fact that it directly evaluates diaphragm function and reflects the work of breathing.

## Supplementary Information

**Additional file 1.**

## Data Availability

The datasets used and/or analysed during the current study are available from the corresponding author on reasonable request.

## Abbreviations

AUROC: Area under the receiver operating characteristic curve
BMI: Body mass index
DE: Diaphragmatic inspiratory excursion
DTF: Diaphragm thickening fraction
ICU: Intensive care unit
IQR: Interquartile range
MV: Mechanical ventilation
NIV: Noninvasive ventilation
PEEP: Positive end-expiratory pressure
PI_MAX_: Maximal inspiratory pressure
PSV: Pressure support ventilation
PTPdi: Transdiaphragmatic pressure–time product
RR: Respiratory rate
RSBI: Rapid shallow breathing index
SBT: Spontaneous breathing trial
SD: Standard deviation
TPIA: Time to peak inspiratory amplitude of the diaphragm
VC: Vital capacity
VT: Tidal volume
