# Supplementary material for: The ratio of respiratory rate to diaphragm thickening fraction for predicting extubation success
Source: BMC Pulm Med. 2023 Apr 4;23:109. doi: 10.1186/s12890-023-02392-w (PMC10071651; doi:10.1186/s12890-023-02392-w)
Supplement: Supplementary file 1 — Additional file 1. [file 12890_2023_2392_MOESM1_ESM.docx]

**Additional File1**

**S1: Local guideline for assessing readiness to wean**

(1) Resolution of disease acute phase for which the patient was intubated

(2) Hemodynamic stable (HR < 140 beats/min^-1^, systolic BP 90–160 mmHg, no or minimal vasopressors)

(3) SaO2 >90% on FIO2 ≤0.4 (or PaO2/FIO2 ≥150 mmHg) and PEEP ≤8 cmH2O

(4) RR/VT <105 breaths/min^-1^/L^-1^

(5) No sedation or adequate mentation on sedation

**S2: Local criteria for failed spontaneous breathing trail**

(1) RR >35 breaths/min^-1^

(2) Increased accessory muscle activity or dyspnea

(3) Systolic BP increased or decreased by ≥20 mmHg

(4) Systolic BP <90 mmHg

(5) HR >140 beats/min^-1^ or increased by >20 beats/min^-1^

(6) Cardiac arrhythmias

(7) PaO2 ≤50 mmHg or SaO2 <90%

(8) pH <7.32

(9) Agitation and anxiety, Depressed mental status, Diaphoresis

**S3: Local criteria for extubation failure**

(1) RR > 30 breaths/min^-1^

(2) Agitation and anxiety

(3) Depressed mental status

(4) Clinical signs of respiratory muscle fatigue or increased work of breathing

(5) SpO2 <90% or PaO2 <60 mmHg

(6) PaCO2 increased by ≥15% from pre-extubation
